# Plasmonic Nanosensors Based on Highly Tunable Multiple Fano Resonances Induced in Metal–Insulator–Metal Waveguide Systems

**DOI:** 10.3390/nano15090686

**Published:** 2025-04-30

**Authors:** Ping Jiang, Yilin Wang

**Affiliations:** 1 School of Science Microelectronics & Data Science, Anhui University of Technology, Maanshan 243002, China; 2 College of Mathematics and Physics, Beijing University of Chemical Technology, Beijing 100029, China

**Keywords:** surface plasmons, plasmonic waveguide, Fano resonance, sensor

## Abstract

We designed and investigated a plasmonic nanosensor with ultra-high sensitivity and tunability, which is composed of a metal–insulator–metal (MIM) waveguide integrated with a side-coupled resonator (SR) and metal baffle. Its high performance is derived from Fano resonance, which is generated by the interaction between the modes of the SR and the baffle, and it can be precisely tuned by adjusting the parameters of the SR. Further investigation based on the incorporation of a side-coupled rectangular-ring resonator (SRR) generates three distinct Fano resonances, and the Fano resonance can be accurately tuned by manipulating the parameters of the resonators within the system. Our proposed plasmonic system can serve as a highly sensitive refractive index nanosensor, achieving a sensitivity up to 1150 nm/RIU. The plasmonic structures featuring independently tunable triple Fano resonances open new avenues for applications in nanosensing, bandstop filtering, and slow-light devices.

## 1. Introduction

Fano resonances originate from the interference between the discrete state and the continuous state in quantum or classical systems with sharp and asymmetric spectral line profiles [1,2,3]. The characteristic of being sensitive to the surrounding medium gives Fano resonances great potential to be applied in sensors [4,5], switching [6], and slow-light devices [7]. Surface plasmon polariton (SPP)-based nanostructures are often used to realize Fano resonances due to their abilities to overcome the diffraction limit of light [8,9] and to manipulate light at the nanoscale [10,11,12,13,14,15,16,17,18], such as plasmonic nanoclusters [14], nanoparticle dimers [15], and metal–insulator–metal (MIM) waveguides [16,17,18].

Particularly, MIM waveguides have received much attention due to their low manufacturing costs, low bending loss, and deep subwavelength field confinements [19,20]. Therefore, it would be possible to realize highly integrated photonic devices by using Fano resonance in MIM plasmonic structures [21]. In the past few years, various kinds of plasmonic MIM structures with different configurations have been designed to generate Fano resonance, such as circle cavities with circle resonators [22], broken symmetry in rectangular cavities [23], and rectangular cavities with slot resonators [24,25]. The design of single or multiple sharp and asymmetrical Fano line profiles, spanning from visible to near-infrared wavelengths, has been realized in these plasmonic MIM structures. Compared with single Fano resonance systems, multiple Fano resonance systems exhibit superior parallel processing capabilities in highly integrated photonic circuits. Moreover, achieving independently tunable Fano resonances is highly desirable for practical optical processing applications. However, it remains challenging to realize the accurate tuning of each Fano resonance in multiple-Fano-resonance systems [26,27,28].

In this work, we theoretically and numerically investigate a plasmonic waveguide system comprising a side-coupled resonator (SR) and a metal–insulator–metal (MIM) waveguide separated by a silver baffle. The ultra-sharp Fano resonance in this system can be precisely manipulated by adjusting the parameters of the SR. Moreover, by incorporating an additional side-coupled rectangular-ring resonator (SRR), the system can be tuned to exhibit triple Fano resonances. These Fano resonances can be accurately tuned through the adjustment of the parameters of the SR and SRR. This plasmonic waveguide system demonstrates great potential as a high-performance refractive index sensor, achieving a sensitivity of 1150 nm/RIU. Given its unique properties, the designed plasmonic waveguide system may find significant applications in nanosensors, bandstop filters, and slow-light devices.

## 2. Structures and Theoretical Analysis

**Figure 1:**
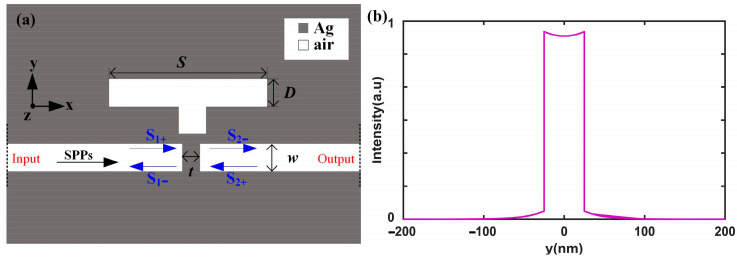
(**a**) Two-dimensional model of the plasmonic configuration composed of an MIM waveguide with a side-coupled resonator and metal baffle. The gray part represents Ag, and the white part represents air. (**b**) The mode profile for the SPPs in the waveguide.

Figure 1a presents a two-dimensional (2D) model of the plasmonic configuration, which consists of a metal–insulator–metal (MIM) waveguide integrated with a subwavelength resonator (SR) and a metallic baffle. The whole structure is infinite in the z direction. In this structure, the SR is positioned directly above the bus waveguide, which is split into two MIM waveguides by a silver baffle. The geometric parameters of the plasmonic waveguide system include the width and length of the resonator (*D* = 50 nm and *S* = 450 nm), the width of the silver baffle (*t* = 20 nm), and the width of the bus waveguide (*w* = 50 nm). In Figure 1a, the gray and white regions represent silver (ε_Ag_(ω)) and air (ε_air_ = 1.0), respectively. The dielectric constant of silver is described by the Drude model [29]: ε_Ag_(ω) = ε_∞_ − ω_p_^2^/(ω(ω + iγ)), where ω is the angular frequency, ω_p_ = 9.1 eV is the bulk plasma frequency, ε_∞_ = 3.7 is the permittivity at infinite angular frequency, and γ = 0.018 eV is the electron collision frequency. These parameters are consistent with the optical constants reported in Ref. [30], where the optical data for silver aligns with experimental results in the wavelength range of 700~1300 nm.

Since the waveguide width is only 50 nm, there is only one basic surface plasmon polariton (SPP) mode, with the mode profile shown in Figure 1b. The introduction of the silver (Ag) baffle and side resonator induce reflections and transmissions within the waveguide, which subsequently interfere with the phase and amplitude of the SPPs propagating through the waveguide. This interference phenomenon is highly complex and significantly alters the line profile of the transmission spectrum of the plasmonic structure, giving rise to an asymmetric and sharply defined Fano resonance line. The transmission spectrum of the system was numerically computed using the Finite Element Method (FEM) implemented in COMSOL Multiphysics 5.4. Specifically, the 2D Electromagnetic Waves, Frequency Domain (ewfd) module was employed to investigate the optical response of the plasmonic structure. In this setup, numeric port 1 (“on”) serves as the input port on the left side, while numeric port 2 (“off”) functions as the output port on the right side. Numeric ports are virtual interfaces used to inject electromagnetic waves in simulations. When a port is “on”, it is active and functions as an excitation port. In addition, the input port is set up as a mode source by mode analysis (the mode profile is shown in Figure 1b). Conversely, when it is “off”, it can only receive the electromagnetic waves in the simulation. Additionally, the boundaries at the domain edges in the y direction were set as the scattering boundary conditions to simulate the conditions at infinity.

To obtain the detailed theoretical analysis of the optical waveguide, we used the multimode interference coupled-mode theory (MICMT) to calculate the transmission spectrum of the plasmonic resonator system [31]. The MICMT equations can be expressed as follows [22,26]:

(1) $$\frac{da_{n}}{dt}=-\left(j\omega_{n}+\frac{1}{\tau_{n0}}+\frac{1}{\tau_{n1}}+\frac{1}{\tau_{n2}}\right)a_{n}+\kappa_{n1}s_{n,1+}+\kappa_{n2}s_{n,2+}$$

(2) $$s_{1-}=-s_{1+}+\sum_{n}\kappa_{n1}^{*}a_{n},\kappa_{n1}=\sqrt{\frac{2}{\tau_{n1}}}e^{j\theta_{n1}}$$

(3) $$s_{2-}=-s_{2+}+\sum_{n}\kappa_{n2}^{*}a_{n},\kappa_{n2}=\sqrt{\frac{2}{\tau_{n2}}}e^{j(\theta_{n2}-\phi_{n})}$$

(4) $$s_{n,1+}=\gamma_{n1}e^{j\varphi_{n1}}s_{1+},s_{n,2+}=\gamma_{n2}e^{j\varphi_{n2}}s_{2+}$$

where $a_{n}$, $\omega_{n}$, and $\tau_{n0}$ are the field amplitude, the resonant frequency, and the decay time of the internal loss in the n-order resonant mode of the side resonator, respectively. $\tau_{n1}$ and $\tau_{n2}$ represent the decay time of the coupling between the MIM waveguide and the n-order resonant mode, respectively. $\theta_{n1}$ and $\theta_{n2}$ are coupling phases between the nth resonator and waveguides, respectively. The coupling between the waveguide mode and the n-order resonant mode is related by the coupling coefficient *κ_n_*_1_ and *κ_n_*_2_, which are determined by Equations (2) and (3). $\phi_{n}$ is the phase difference from the input port to the output port of the nth resonant mode. $s_{i+}$ (i=1,2) and $s_{i-}$ (i=1,2) represent the input and output field amplitudes in the waveguide, respectively. According to the symmetry of the MIM waveguide, and due to the fact that the SPPs are only input into the waveguide from the left side, we set $s_{2+}=0,$ $\gamma_{n1}=\gamma_{n2}\approx 1,$ $\tau_{n1}=\tau_{n2}=\tau_{n},$ and $\theta_{n1}=\theta_{n2}$. The transmittance of the plasmonic resonator system is calculated as follows:

(5) $$T={\left|t\right|}^{2}={\left|\frac{s_{2-}}{s_{1+}}\right|}^{2}={\left|\sum_{n}\frac{2e^{j\varphi_{n}}}{-j(\omega -\omega_{n})\tau_{n}+2+\frac{\tau_{n}}{\tau_{n0}}}\right|}^{2},\varphi_{n}=\varphi_{n1}+\phi_{n}$$

The resonance wavelength in the plasmonic resonator system should satisfy the resonance condition [32,33]:

(6) $$\frac{d\lambda }{dL_{eff}}=\frac{2n_{eff}}{N-\varphi /\pi }\;(N=1,2,\ldots )$$

where *n_eff_* is the effective index of the SPPs. d*λ* and d*L_eff_* are the shift in the resonance wavelength and the change in the resonator length, respectively. The equation indicates that d*λ* is linear with d*L_eff_* and is proportional to *n_eff_*.

## 3. Simulation Results and Discussions

**Figure 2:**
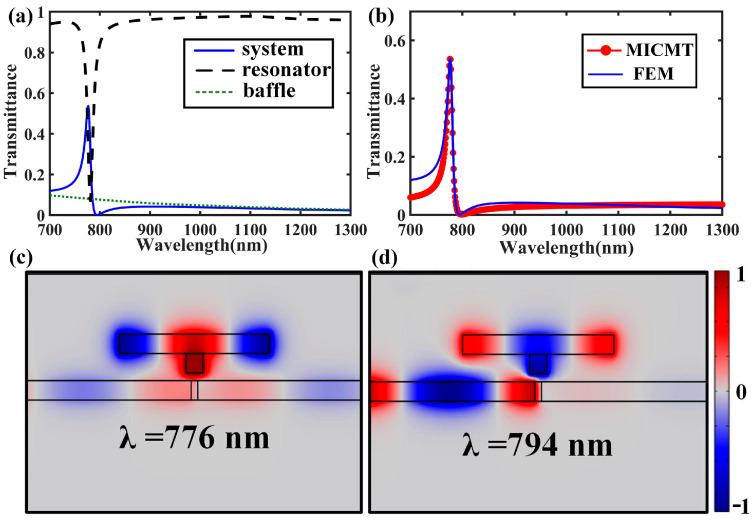
(**a**) Transmission spectra of the proposed plasmonic system, single side resonator, and the metallic baffle. (**b**) Transmission spectra of the proposed plasmonic structure calculated by the MICMT (red circle line) and FEM (blue line) methods. (**c**) The normalized field distributions for the z-component of the magnetic field (H_z_) at the peak wavelength of *λ* = 776 nm (maximum Fano resonance). (**d**) H_z_ distributions at a dip wavelength of *λ* = 794 nm (minimum Fano resonance).

As shown in Figure 2a, there was a sharp asymmetric Fano resonance line shape in the transmission response of the system [18]. The transmission spectra for the single side resonator and metallic baffle were also calculated, from which we can see that the transmission spectrum for the baffle exhibited a broad line, while the counterpart for the single resonator exhibited narrow dips located at almost the same wavelength of the Fano resonance. Thus, we can conclude that the Fano resonance of the whole system originated from the interference between the broad and narrow transmission spectra. The MICMT was used to fit the transmission response of the plasmonic waveguide system, and the results are shown in Figure 2b with the red dot line. The complex amplitude transmission coefficient of each resonance mode was set as $t_{n}=2e^{j\varphi_{n}}/\left[-j(\omega -\omega_{n})\tau_{n}+2+\tau_{n}/\tau_{n0}\right].$ There was only one strong resonant mode ($t_{1}$) in the simulation results. The constant ($t_{0}$) was the contribution of the complex amplitude transmission coefficient of the other resonant modes. In this case, the transmittance can be expressed as $T={\left|t_{0}+t_{1}\right|}^{2}.$ We obtained the parameters in the formula by curve fitting, which were $t_{0}=0.2,$ $\varphi_{1}=0.64\pi ,$ $\tau_{1}=150$ fs, and $\tau_{10}=250$ fs, respectively. In terms of the values of the fitted parameters, for example, *τ*_10_ and *τ*_1_ represent the decay time of the coupling between the MIM waveguide and the first resonant mode, which determines the transmission intensity and line width of the Fano resonance. The smaller the value of *τ*_10_ and *τ*_1_, the stronger the coupling strength, which will result in higher-intensity Fano resonance in the transmission. It can be seen from Figure 2a that the computation result obtained from MICMT (red dot line) was consistent with the transmittance simulated by the FEM (blue line).

To further analyze the underlying mechanisms of the Fano resonance in the plasmonic waveguide system, the normalized field distributions for the z-component of the magnetic field (H_z_) at *λ* = 776 nm and *λ* = 794 nm are shown in Figure 2b and Figure 2c, respectively. We used TM*_nm_* to classify the resonant modes, where *m* and *n* are integers, representing the resonant orders in the x direction and y direction, respectively. Obviously, the energy was almost confined in the SR for the TM_20_ mode. Therefore, the main response of the transmittance was determined by the SR. The distribution of H_z_ at the output port also showed that the intensity of H_z_ was stronger at *λ* = 776 nm, while it was weaker at *λ* = 794 nm. This led to the maximum and minimum values of the Fano resonance in the spectrum.

**Figure 3:**
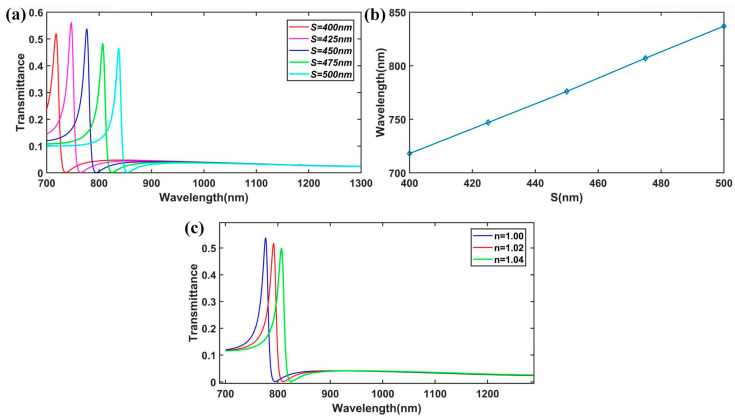
(**a**) The FEM results of the transmission spectra for different values of *S*. (**b**) The location of the peak of the Fano resonance varied with the lengths of *S*. (**c**) Transmission spectrum of the different media (*n* = 1.00~1.04).

Then, we investigated the influence of the length of the subwavelength resonator (SR) on the transmission spectrum. The transmission spectra of the plasmonic waveguide system for various lengths of *S* (*D* = 50 nm, *t* = 20 nm, and *w* = 50 nm) are presented in Figure 3a. When the length *S* varied from 400 nm to 500 nm in increments of 25 nm, the resonant wavelength of the Fano resonance shifted from *λ* = 718 nm to *λ* = 837 nm. This shift exhibited a linear redshift trend as *S* increased (Figure 3b), which is in excellent agreement with the predictions made by Equation (6). This linear movement of the peak location enables the effective tuning of the Fano resonance to the desired wavelength.

Furthermore, the transmittance of the Fano resonance dropping sharply from the peak to the valley in the spectra provided a highly sensitive spectral response to changes for different surrounding media. Figure 3c illustrates the transmission spectra of the plasmonic structure for different refractive indices *n* of the insulator part (waveguides and the subwavelength resonator, SR) when the sensing analyte was arranged in the waveguides and the resonator. It is evident that the resonant wavelength exhibited a redshift with the increase in the refractive index, consistent with the results described in Equation (6). To evaluate a sensor’s performance, the sensitivity is often evaluated. The sensitivity (nm/RIU) of each Fano resonance is defined as d*λ*/∆*n,* where RIU is the refractive index unit [34]. From Figure 3c, we can calculate the sensitivity of the plasmonic structure, which was approximately 750 nm/RIU. This high sensitivity makes it a promising candidate for refractive-index-sensing applications.

## 4. Transmission Properties of the Extended Plasmonic Structure

**Figure 4:**
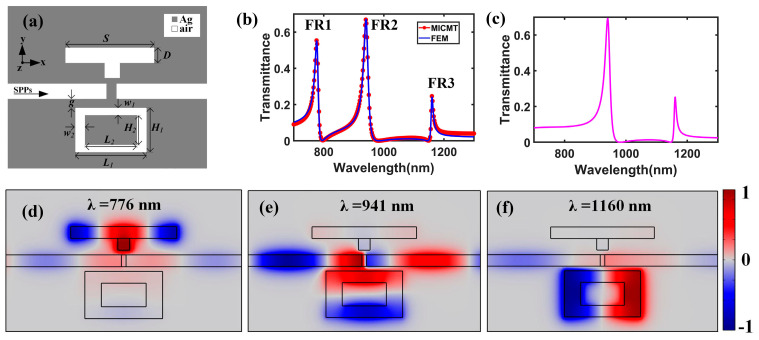
(**a**) Schematic diagram of the improved plasmonic structure by adding a side-coupled rectangular-ring resonator. (**b**) Transmission spectra of the improved plasmonic structure based on the MICMT (red circle line) and FEM (blue line) methods. (**c**) The spectrum of the SRR system without the side-coupled resonator. The distribution of the normalized magnetic field (H_z_) of (**d**) FR1 at 776 nm, (**e**) FR2 at 941 nm, and (**f**) FR3 at 1160 nm.

To realize multiple Fano resonances, an SRR with a coupling distance *g* = 20 nm was added on the opposite side of the waveguide, as depicted in Figure 4a. The main parameters of the SRR were the outer wall width (*L*_1_) and height (*H*_1_), the inner wall width (*L*_2_), and height (*H*_2_). *H*_1_ and *H*_2_ were set to be 200 nm and 100 nm, respectively. Figure 4b shows the corresponding transmission spectrum (blue line) of the improved plasmonic structure with *L*_1_ = 330 nm, *L*_2_ = 190 nm, *w*_1_ = 50 nm, and *w*_2_ = 70 nm. Compared to the transmittance spectrum of the subwavelength resonator (SR) system, the wavelength of the first Fano resonance (FR1) remained unchanged, while two new Fano line shapes (FR2 and FR3) emerged in the spectrum. We also used MICMT to fit the transmission spectrum of the improved plasmonic waveguide system with $T={\left|t_{0}+t_{1}+t_{2}+t_{3}\right|}^{2}$. According to the results of curve fitting, we obtained $t_{0}=0.23,$ $\varphi_{1}=0.575\pi ,$ $\tau_{1}=140$ fs, $\tau_{10}=165$ fs, $\varphi_{2}=0.555\pi ,$ $\tau_{2}=125$ fs, $\tau_{20}=265$ fs, $\varphi_{3}=-0.675\pi ,$ $\tau_{3}=750$ fs, and $\tau_{30}=415$ fs. As denoted by the red circle line in Figure 4b, the theoretical results were consistent with the FEM simulation results. Figure 4c shows the spectrum of a system complementary to the first configuration without the side-coupled resonator. There were only FR2 and FR3, as expected. Figure 4d–f show the distribution of the normalized magnetic field of FR1, FR2, and FR3, respectively. The normalized magnetic field distribution at FR1 was still confined in the SR, which indicates that it was not affected by the SRR. For FR2, the energy was mainly distributed in the transverse waveguide of the SRR, indicating that the resonance wavelength of FR2 was affected by *L*_1_, *H*_1_, *L*_2_, and *H*_2_. In addition, the magnetic field energy at the resonance wavelength of FR3 was concentrated at the ring corners of the SRR, which indicates that the resonant wavelength of FR3 was mainly affected by *L*_1_ and *H*_1_. Therefore, the resonance wavelengths of FR2 and FR3 were affected by the SRR and unrelated to the SR. Thus, we can manipulate the resonant wavelength of FR1 independently by tuning the parameters of the SR or manipulate the resonant wavelengths of FR2 and FR3 by changing the parameters of the SRR.

**Figure 5:**
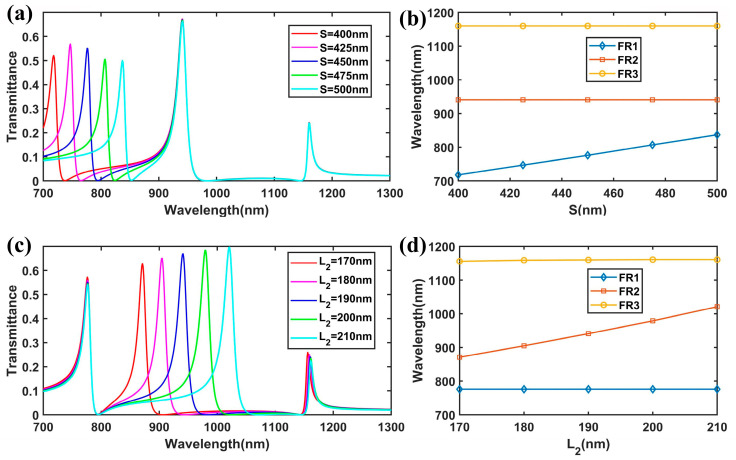
(**a**) The FEM results of the transmission spectra for different values of *S*. (**b**) The variations in the peak wavelength of the Fano resonances with the change in the length of *S*. (**c**) Transmission spectra for different lengths of *L*_2_. (**d**) The variations in the peak wavelength of the Fano resonances with the change in the length of *L*_2_.

Successively, we tuned the transmission spectrum of the improved plasmonic structure by manipulating the parameters of its components. Firstly, we studied the shift in the resonant wavelengths when *S* changed from 400 nm to 500 nm, as shown in Figure 5a. We found that the resonant wavelength of FR1 had a redshift, while the resonant wavelengths of FR2 and FR3 remained fixed. Figure 5b shows a more detailed analysis of the variation trend for FR1, FR2, and FR3, where the resonant wavelength of FR1 exhibited a linear redshift from 718 nm to 837 nm with the increase in *S*. Next, we set the length *S* to 425 nm and adjusted the width *w*_2_ by varying *L*_2_. The transmission responses for different *L*_2_ values are shown in Figure 5c (*L*_1_ = 330 nm). The resonant wavelength of FR2 had a red shift from 871 nm to 1021 nm, while the resonance wavelengths of FR1 and FR3 were almost unchanged, which can also be identified in Figure 5d. Herein, we achieved the independent tuning of FR1 and FR2. This independent tunability of the Fano resonances holds significant value for the development of high-performance nanosensors.

**Figure 6:**
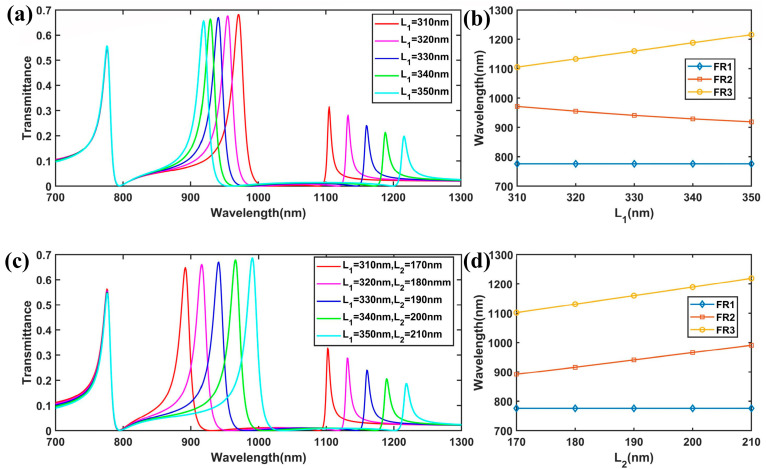
(**a**) The FEM results of the transmission spectra for different values of *L*_1_. (**b**) The variations in the peak wavelength of the Fano resonances with the change in the length of *L*_1_. (**c**) Transmission spectra for different lengths of *L*_1_ and *L*_2_. (**d**) The variations in the peak wavelength of the Fano resonances with the change in the lengths of *L*_1_ and *L*_2_.

Then, we controlled *w*_2_ by changing *L*_1_ (*L*_2_ = 190 nm). Figure 6a,b show the transmission spectra of the improved plasmonic structure with different values of *L*_1_. The results show that the resonant wavelength of FR1 remained unchanged, while FR2 exhibited a nonlinear blue shift from 971 nm to 919 nm. As *L*_1_ varied from 310 nm to 350 nm, the width w_2_ changed from 60 nm to 80 nm, and the effective refractive index decreased for the mode distributed along the y direction in the SRR. Consequently, FR2 exhibited a nonlinear blueshift with the increase in *L*_1_, a trend that can be anticipated from Equation (6). On the contrary, the resonant wavelength of FR3 exhibited a linear redshift from 1105 nm to 1216 nm. This is because when *L*_1_ increased, the outer wall of the SRR increased linearly, while the effective refractive index at the corner remained unchanged. Finally, we kept *w*_2_ = 70 nm unchanged while changing *L*_1_ and *L*_2_. With the increase in *L*_1_ and *L*_2_, FR2 exhibited a redshift from 892 nm to 991 nm, and FR3 exhibited a redshift from 1102 nm to 1218 nm, as shown in Figure 6c,d. The above results indicate that the improved plasmonic waveguide system can serve as a band stop filter for different frequency bands due to the tunable ability of the multiple Fano resonances and the ultra-low broad transmission dip.

**Figure 7:**
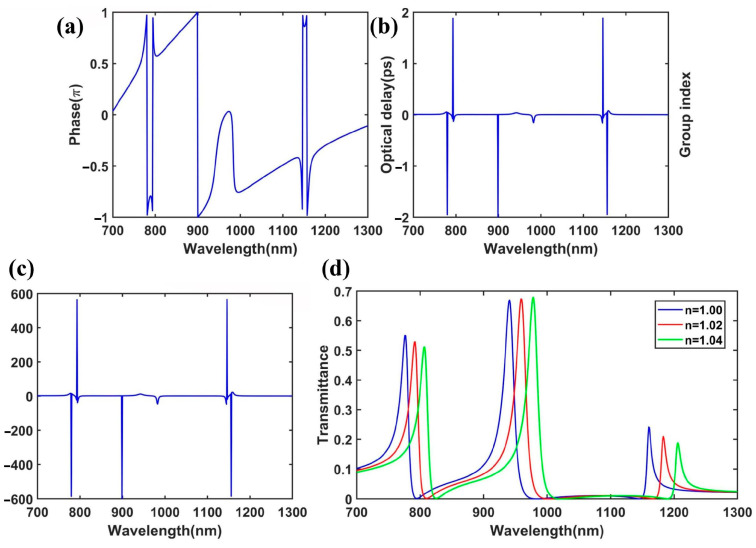
(**a**) Transmission phase shift, (**b**) optical delay line, and (**c**) group index of the plasmonic system. (**d**) Transmission spectra of the plasmonic system with different refractive indices of the dielectric in the waveguides and cavity, with *n* = 1.00 (blue line), *n* = 1.02 (red line), and *n* = 1.04 (green line).

In addition, the group index *n_g_*, which describes the slow-light effect of the Fano resonances, was also investigated using Equation (7) [35,36]:

(7) $$n_{g}=\frac{c}{L}\cdot \frac{d\varphi (\omega )}{d\omega }$$

where *c* is the speed of light in a vacuum. *L*, φ(ω), and ω represent the length of the system, the transmission phase shift, and the resonant frequency, respectively. φ(ω) can be directly calculated by the phase for complex values of H_z_, which can be defined as *φ*(*ω*) = arctan (imag(H_z_)/real(H_z_)). imag(H_z_) and real(H_z_) are the image and real part of H_z_, respectively. The simulated transmission phase shift from the input to the output is shown in Figure 7a. In addition, the corresponding optical delay and group index can be calculated by Equation (7), as illustrated in Figure 7b,c. Obviously, the optical delay and group index dramatically increased near the Fano peaks due to the rapid phase change at the Fano resonances. We observed that the maximum group index was about 600 near the Fano peaks. Therefore, the plasmonic system with a large group index holds great potential for applications in ultra-compact slow-light devices.

Similarly, we investigated the sensing performance of the improved plasmonic waveguide system. We calculated the transmission spectra of the structure with different refractive indexes, as depicted in Figure 7d. The sensitivity values of the Fano resonances were about 800 nm/RIU for FR1, 900 nm/RIU for FR2, and 1150 nm/RIU for FR3. The sensitivity for FR1 was higher than that in the SR (750 nm/RIU). This may be due to the change in the refractive index enhancing the influence of the SRR cavity modes on the SR cavity modes. Thus, the improved plasmonic waveguide system could have several applications in nanosensors.

## 5. Conclusions

In summary, we introduced a nanosensor comprising a subwavelength resonator (SR) and a metal–insulator–metal (MIM) waveguide separated by a silver baffle. The transmission characteristics of this plasmonic structure were thoroughly analyzed and investigated. The simulation results demonstrate that the ultra-sharp Fano resonances can be effectively manipulated by adjusting the parameters of the SR. Furthermore, by incorporating a split-ring resonator (SRR) into the plasmonic waveguide system, three distinct Fano resonances could be achieved. Each Fano resonance could be accurately tuned by modifying the parameters of the improved plasmonic structure. The enhanced design exhibited a sensitivity of approximately 1150 nm/RIU, making it a highly effective nanosensor. Our proposed structures hold significant potential for applications in high-performance nanosensors, slow-light devices, and bandstop filters.

## Data Availability

Data are contained within the article.
